# Deqi Sensation in Placebo Acupuncture: A Crossover Study on Chinese Medicine Students

**DOI:** 10.1155/2013/620671

**Published:** 2013-07-29

**Authors:** Zhao-hui Liang, Chang-cai Xie, Zi-ping Li, Xiao-ping Zhu, Ai-ping Lu, Wen-bin Fu

**Affiliations:** ^1^The Second Affiliated Hospital of Guangzhou University of Chinese Medicine, Guangzhou 510120, China; ^2^School of Chinese Medicine, Hong Kong Baptist University, Kowloon Tong, Hong Kong

## Abstract

*Objective*. To evaluate the similarity of deqi sensation of real and noninvasive placebo acupuncture in healthy people with knowledge of Chinese medicine. *Methods*. In a crossover design, volunteers recruited from Chinese medicine college students were randomized to two groups to receive two phases of intervention with a one-week washout interval. In Group A, the participants were firstly treated by real acupuncture and then by sham needle, and the treatment sequence was reversed in Group B. VAS for pain intensity and deqi sensation was evaluated as outcomes. *Results*. Sixty-three volunteers were recruited and 60 were included and finished the study. In Group A, VAS was higher in Phase I than in Phase II (*P* = 0.017). Only treatment methods were selected as factor to VAS difference (*P* = 0.046) in ANOVA test. More positive deqi was reported in Group A in Phase I when treated by real acupuncture (*P* = 0.039), but the difference was not significant in Phase II (*P* = 0.301). *Conclusion*. The noninvasive placebo acupuncture device can effetely simulate the deqi sensation as real acupuncture, but it is less likely to evoke the active effect of deqi in real practice. This trial is registered with Chinese Clinical Trial Registry: ChiCTR-ORC-09000505.

## 1. Background

Deqi or acupuncture sensation is a unique phenomenon in the practice of acupuncture. In a previous report, deqi was described as the combination of various sensations, for example, aching, dullness, heaviness, numbness, radiating, tingling and spreading, and so forth [1]. The mechanism of deqi has been studied and is characterized as the stimulus conducted by a wide spectrum of nerve fibers from the perspective of neurophysiology [2]. It is believed that the effect of acupuncture depends on the due acquisition of deqi, and this idea was supported by acupuncture analgesia in which the analgesic effect can be acquired only when deqi is felt by patients [3]. However, deqi is not always stressed in clinical studies. The main clinical study method of acupuncture is randomized control trail (RCT); thus, it must follow the common methodology principles, that is, randomization, appropriate control, blinding, and repeatability. Though deqi is an important factor for effectiveness, it is difficult to duplicate from one patient to another because of the difference in manipulation by practitioners and the variety of patients' response even to the same dosage of deqi. A study reported that Chinese patients had more intensive sensation of deqi than US patients, hence, leading to better therapeutic effects [4]. Therefore, a reliable control treatment capable of shielding deqi is crucial to the success of acupuncture RCTs.

In a brief review of six clinical studies during the nearest decade in which deqi was clearly reported, three of them with a total of 851 cases had negative results [5–7], compared to the rest three studies with positive outcomes with 308 cases in total [8–10]. Besides the sample size, the use of sham acupuncture is another key factor for effectiveness. Two of the studies reported the use of noninvasive sham needle (Park's sham device [7] and Streitberger's device [6]) with observed patients cumulated to 258. Since placebo control was introduced by Vincent et al. in 1995 as a rigorous and consensus approach to enhance the methodological quality of acupuncture trials, a series of placebo control methods have been applied in acupuncture trials. For instance, sham needling invasive to subcutaneous or dermal tissues at non-acupoints [10, 11], noninvasive sham needle with a blunt tip, sham laser acupuncture [12], and sham electrode on acupoints without electricity [13]. The authors found that there is a trend that the newer the report is, the more confirmed that deqi will attribute to the active interventional effect. Therefore, in our opinion, an ideal placebo device should refrain the confounding of deqi during its intervention. The previous reports indicated that deqi is generated by the stimulus to the nerve system; hence, the invasive sham acupuncture methods will more or less produce active effects. Based on this assumption, we assume that the less the intensity of deqi is, the less confounding effect will contaminate the study outcome. And the noninvasive sham needles are believed to produce the least quantity of deqi because they do not penetrate skin hence cause the least activation of neural receptors. And this assumption founds the rationale basis of the study.

In this work, we presented a crossover study to evaluate the deqi sensation and placebo effect of a noninvasive sham needle device. The participants are college student volunteers specializing in Chinese medicine or acupuncture from Guangzhou University of Chinese medicine. They have comprehensive knowledge on acupuncture and deqi; thus, it is hopeful to reveal whether noninvasive sham devices are able to imitate deqi as placebo control in acupuncture RCTs. The study is reported in accordance with CONSORT 2010 statement [14] and STRICTA 2010 extension for acupuncture [15]. 

## 2. Design and Methods

### 2.1. Trial Design

The study was designed as a randomized, two-arm, single-blind, and crossover clinical trial. The study subjects were randomly allocated to two groups (Group A and Group B) and then sequentially received two phases of intervention of real acupuncture and noninvasive sham needle or vice versa. The two phases were separated by a one-week washout interval to eliminate the residual effect by the former phase. The outcomes included the quality of deqi sensation and pain intensity. The study objective is to evaluate the similarity of deqi sensation of real and placebo acupuncture in healthy people with comprehensive Chinese medicine and acupuncture knowledge.

### 2.2. Ethics Review and Informed Consent

The study protocol was reviewed and approved by the Ethics Committee of Guangdong Provincial Hospital of Chinese Medicine (no. 2008GL-27), and registered in the Chinese Clinical Trial Registry (no. ChiCTR-ORC-09000505), which is a primary registry in the WHO registry network (http://www.chictr.org/en/). All patients were required to provide informed consent.

### 2.3. Inclusion Criteria

Participants were included only when they met the following inclusion criteria:

- age 20 to 23 years (both males and female are included),
- physically and mentally healthy confirmed by a physician,
- having studied Chinese medicine and acupuncture for at least two years,
- having finished the courses of basic Chinese medicine and acupuncture,
- having acupuncture experience beforehand,
- no acupuncture therapy within the 3 months prior to study entry,
- good protocol compliance and agreeing to sign an informed consent document.

### 2.4. Exclusion Criteria

Participants would be excluded if they had one of the following conditions:

- skin lesions on the surface of selected acupoints,
- skin diseases affecting the skin surface of selected acupoints,
- complaint of acute or chronic pain,
- use of analgesics or psychotropic drugs,
- pregnancy or lactation,
- rejection or fear of acupuncture,
- considered not suitable to participate the trial by the researchers.

### 2.5. Sample Size and Randomization

As no data can serve as referred parameters, we assume that the sample size is 60 in total. The participants were randomized to two groups to, respectively, receive noninvasive sham needle and real acupuncture in the reverse order. The random allocation sequence was generated by an independent statistician with the use of SAS software (SAS Institute, Cary, NC, USA, version 9.2 (TS2 M3), licensed to THE SECOND CLINICAL COLLEGE OF GUANG ZHOU U, Site 11201875). The allocation codes were sealed into 60 opaque envelopes and all participants would be allocated in accordance with the randomized code of the corresponding envelopes.

### 2.6. Device and Intervention

**Figure 1:**
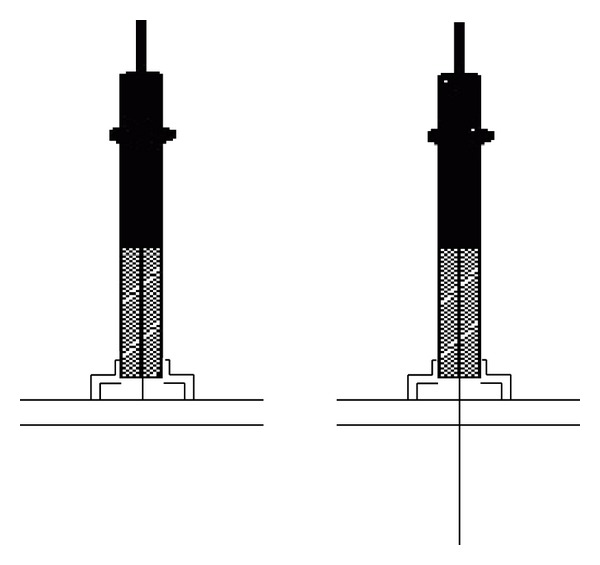
The mechanism of noninvasive sham needle.

There were two kinds of interventions in the study, that is, the noninvasive sham needle and real acupuncture. The instruments were also known as Park Sham Device set produced by DongBang Acuprime (http://www.acuprime.com/). The Sham device is composed of a blunt sham needle in two plastic tubes one slides into the other connected with a sticky opaque base on the skin. When the sham needle is inserted, it only hits the skin surface without penetration; thus, it is supposed to only produce the pinprick feeling but without active acupuncture effects. On the other hand, when the real needles pass through the tube, they will penetrate the skin and thus produce active effects. The sham needles and real needles in this study were 0.3 mm × 40 mm in size. The practitioners can manipulate the handle for deqi after the needles are inserted. Since the device base is opaque, the subject is unable to tell whether the needle penetrates the skin or not (see Figure 1). The device is registered in the European Union as CE 0120 and in the US FDA as License No. 263.

### 2.7. Blinding and Point Selection

**Figure 2:**
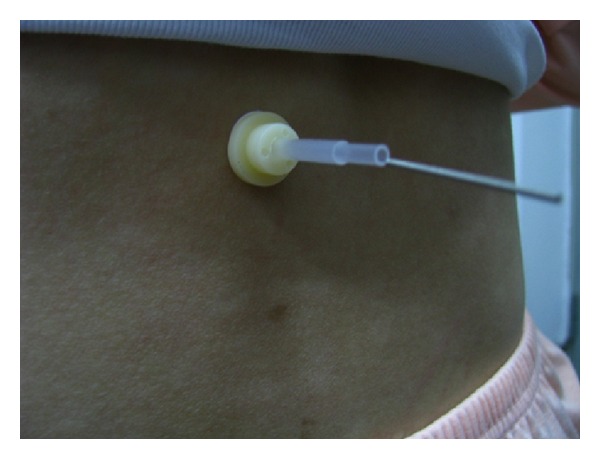
Sham acupuncture.

**Figure 3:**
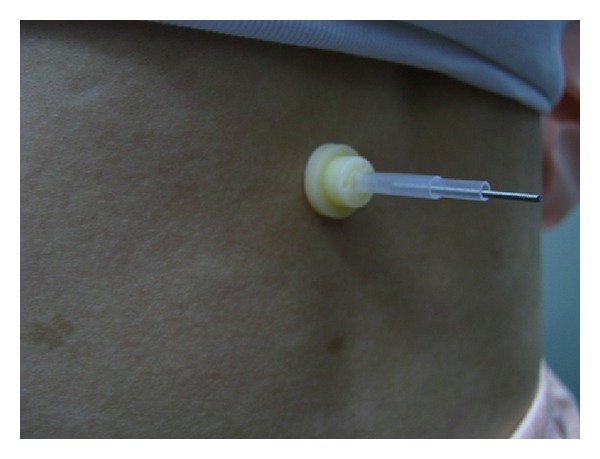
Real acupuncture.

As the intervention must be performed by an acupuncturist and needle manipulation is prerequisite for deqi, the allocation concealment to acupuncturist is not feasible in this study. Thus, we applied a single-blind design in which both the volunteer participants and statisticians were blinded to group allocation till the final data analysis was finished. The selected point was Shenshu (BL 23) bilateral whose location followed the WHO standards [16]. The acupoints are located on the back; thus, the participants could not observe the needle and manipulation during the intervention. Therefore, we concluded that the blinding was effective in this study to all participants. See Figures 2 and 3.

### 2.8. Intervention Period and Procedure

**Figure 4:**
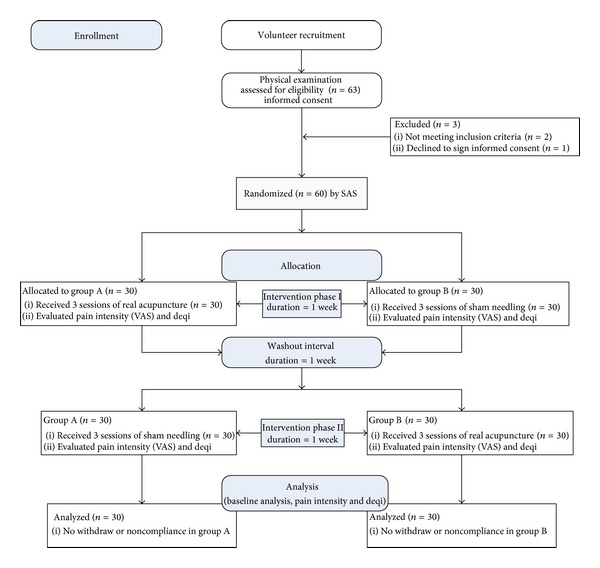
Flowchart of study procedure.

The intervention consisted of two phases: sham needle intervention and real acupuncture. And there was a one-week washout interval between the phases to eliminate the residual effects in the preceding phase. A single phase was one week during which the participants received three sessions (20 minutes per session) of intervention every other day after being included and allocated to a group. In group A, the participants received real acupuncture at first and then sham needle intervention after the washout interval; in group B, the participants received sham needle intervention at first and then real acupuncture after the washout interval. The interventions were performed indoors with the average temperature of 25°C. After local disinfection by medical alcohol, the needles were inserted and manipulated for 30 seconds to induce deqi in both groups. The devices were retained in the acupoints for 5 minutes. All interventions were performed by an identical practitioner from the Acupuncture Department of Guangdong Provincial Hospital of Chinese Medicine. The intensity of pain and deqi sensation were evaluated in the end of both phases. The study procedure is illustrated in the flowchart (see Figure 4).

### 2.9. Outcomes for Evaluation

Both pain intensity and deqi sensation were assessed in this study. Pain intensity was measured on a visual analog scale (VAS). And deqi sensation was evaluated in two dimensions: the deqi feeling (including dullness, heaviness, numbness, radiating, and tingling) and the feeling of skin penetrated. A questionnaire was used for the assessment of deqi feeling, in which a positive response to any one of the perceptions including dullness, heaviness, numbness, radiating, and tingling would be considered as deqi. Because the deqi feeling is related to efficacy in real treatment while the feeling of skin penetrated is related to the concealment or the effect of blinding, they should be assessed separately. The outcome data were collected by patient-reported outcome (PRO) method, with the questionnaire being completed by the participants themselves with necessary instruction from the researchers.

### 2.10. Statistical Analysis

EpiData (version 3.1) was used for data entry and SPSS (version 16.0; SPSS Inc., Chicago, IL, USA) for statistical analysis. The analytic strategy included the descriptive analysis on demographic characteristics of the participants and the outcome of pain and deqi. The group differences were tested by *t*-test for continuous variables, the Mann-Whitney test (two independent samples) or Wilcoxon test (two related samples) for continuous variables not complying with normal distribution, and the Chi-square test for categorical variables. The significance level is set at *α* = 0.05, two-sided. The intention-to-treat (ITT) strategy would be applied if there were withdraws or noncompliance from the study.

## 3. Results

### 3.1. Volunteer Recruitment

Recruitment leaflets were distributed on the two campuses of Guangzhou University of Chinese Medicine for volunteer participants who were junior or senior student of Chinese medicine or acupuncture. The enrolled participants are given a physical examination by doctors from Guangdong Provincial Hospital of Chinese Medicine to confirm their healthy status. The participants were included only after they met the inclusion criteria and had signed an informed consent document. In the study, 63 volunteers were recruited. Two of them were later identified not meeting the inclusion criteria and one refused to sign the informed consent document. Hence, 60 were finally included in the trial and finished the observation without any withdraw. The participant flow is illustrated in Figure 4 of Section 2.8.

### 3.2. Baseline Demographic Data

A total of 63 participants were recruited in which 60 were eventually included and finished the study. There were 8 males and 22 females in Group A whose average age was 23.13 ± 0.62, and there were 4 males and 26 females in Group B whose average age was 22.93 ± 0.58. No statistical difference was found in sex and age between the two groups. (See Table 1).

### 3.3. Pain Intensity

The pain intensity caused by real needle insertion and sham needle pricking was measured by visual analog scale (VAS) in the end of each intervention phase. The ANOVA test for crossover design showed that only treatment methods contributed to the VAS difference in the study (*P* = 0.046). The between-group comparison showed the VAS scores had no statistical difference between groups in both Phase I (*P* = 0.053) and Phase II (*P* = 0.282). And in within-group comparison, the VAS score of Group A in intervention Phase II was higher than in intervention Phase I (*P* = 0.017), while the VAS scores in Group B had no statistical difference between Phase I and Phase II (*P* = 0.690). (See Tables 2 and 3).

### 3.4. Report of Deqi Feeling

Deqi is a subjective feeling which is described as a serial of sensations such as dullness, heaviness, numbness, radiating, and tingling. Thus we designed a questionnaire to inquire the participants whether dullness, heaviness, numbness, radiating, or tingling was perceived during the intervention based on their background knowledge and understanding. A positive response to any of the above sensations was considered as deqi. If all responses were negative, deqi was considered absent. In Phase I, 19 participants (63.3%) reported deqi in Group A compared to 11 participants (36.7%) in Group B, in which the difference was statistically significant (*P* = 0.039). In Phase II, 12 participants (40.0%) reported deqi in Group A compared to 16 participants (53.3%) in Group B, in which the difference was not statistical significant (*P* = 0.301). In within-group comparison between Phase I and Phase II, the reports of deqi had no statistical significance in both Group A (*P* = 0.143) and Group B (*P* = 0.332). (See Table 4).

### 3.5. Report of Skin Penetrated Feeling

Among the reports of skin-penetrated feeling, 21 (70.0%) participants in Group A and 23 (76.7%) in Group B gave a positive response in Phase I, and 25 (83.3%) participants in Group A and 23 (76.7%) in Group B gave a positive response in Phase II. The between-group difference was not statistically significant (*P* = 0.559 in Phase I & *P* = 0.519 in Phase II). In within-group comparison between Phase I and Phase II, the reports of skin penetrated feeling had no statistical significance in both Group A (*P* = 0.424) and Group B (*P* = 1.000). (See Table 5).

### 3.6. Safety Evaluation

The adverse events (AE) were local bleeding, numbness, and radiating on the acupoints being needled. In this study, 5 cases of local bleeding were reported in Group A and other 3 cases were reported in Group B. The local bleeding was stopped within one minute when pressed by medical cotton. There were 9 complaints of local sustained numbness and radiating after the needle devices were withdrawn in Group A and 7 in Group B. The symptoms were effectively controlled within 30 minutes when applied with hot towels on the local skin. 

## 4. Conclusion and Discussion

In this study, we designed a randomized, two-arm, single-blind, and crossover clinical trial to evaluate the deqi sensation of a noninvasive sham needle device and to explore the influence of deqi on placebo effect in RCTs with placebo control of acupuncture. The concealment and placebo effect of sham acupuncture have been assessed in a variety of studies outside China on subjects who were naïve to acupuncture and Chinese medicine [17] and who had acupuncture experience [18]. The currently notion is that the deqi sensation is more than pain [18], and it is a comprehensive acupuncture experience composed of perception of various dimensions. White et al. introduced the 17-item Southampton Needle Sensation Questionnaire to measure deqi which contains 7 items for pain sensations (i.e., “Aching deqi”) and other 7 items for nonpain sensations (i.e., “Tingling deqi”) [19]. Kim et al. developed the 19-item Acupuncture Sensation Questionnaire (ASQ) to measure deqi from real acupuncture experiences [20]. The similarity of these two questionnaires is that the sensations of deqi are both deconstructed to pain-related sensations and nonpain sensations despite the inconsistency in other aspects. Thus, it becomes the consensus on common understanding of deqi in the nearest decade. However, Deqi sensation is not simply pain but consists of a variety of acupuncture experiences and perceptions. The previous questionnaires only include the understanding and perceptions of Deqi by ordinary people, but they ignore the fact that Deqi is more than pain or unhappy feelings: it also directly affects the efficacy of acupuncture treatment. We thus organized this study in which participants were medical students of Chinese Medicine with profound understanding of Chinese medicine acupuncture. In the deqi assessment, the sensation was summarized into two questions: the overall feeling of deqi and the feeling of skin penetrated. The participants were required to make a decision based on their comprehensive knowledge of Chinese Medicine which covered all factors of deqi.

On the other hand, placebo device was advocated in acupuncture studies since 1995 [21]. An ideal placebo should comply with two principles: similarity in appearance and manipulation and production of no active effects [22]. Streitberger and Kleinhenz introduced the first sham device (Streitberger's device) in 1998 [23], but its placebo and concealment effect was challenged by later studies because the sham device was likely to be distinguished by subjects or even had superior effects over real acupuncture [24–26]. Park invented another sham device (Park Sham Device) in 2009 [27]. And it was considered as a credible sham control in both acupuncture naïve and experienced population [28]. Both Streitberger's device and Park Sham Device belong to noninvasive or nonpenetrating sham needle, which is composed of a blunt tip sham needle, a tube for needle insertion, and an opaque base sticky on the acupoint skin. The real or sham needle can be inserted through the tube and manipulate in the opaque base without being distinguished by participants. However, recent acupuncture trials in which sham needles were applied as placebo control still indicated noninferior efficacy of placebo acupuncture compared to real acupuncture [29, 30]. The outcome may attribute to the complexity of acupuncture treatment in which various factors contribute to the overall outcome; hence, the placebo devices are likely to affect actively except for their nonspecific (i.e., placebo) effect. These studies [17–20, 23, 27, 28] focused on evaluating the concealment of placebo device, but the issue that whether and to what extent the placebo devices affect actively was not specifically answered. Furthermore, how deqi sensations affect the treatment outcome and whether needle manipulation attributes to the efficacy are controversial issues in current studies.

Our work is to evaluate the deqi sensation stimulated by noninvasive sham needle device in college students of Chinese medicine. Deqi is a unique feeling induced by acupuncture, and it is believed that the perception of deqi is based on profound understanding and experience of Chinese medicine and acupuncture. Compared to ordinary people with acupuncture experience, our volunteer participants have been trained in Chinese medicine for at least two years; hence, their knowledge and experience are adequate to perceive deqi without concept deconstruction. In addition, pain is an inseparable component of deqi sensation and its intensity is related to both deqi and concealment; therefore, pain was quantitatively measured by VAS combined with the feeling of skin penetration as the assessment for concealment. 

The result indicated that the noninvasive sham device produced more pain in intensity than real needle for its mean of VAS score was higher than real acupuncture in Phase I intervention though it is not statistically significant (*P* = 0.053). The ANOVA test for crossover study indicated only different treatment methods contributed to the VAS difference in the study (*P* = −0.046). The difference in VAS might also be affected by gender because more females were concluded in the study than men, and women are considered more sensitive to pain. For the assessment of deqi feeling, more participants reported deqi when they were treated and manipulated by real acupuncture. However, the volunteers could not distinguish the deqi feeling induced by real or sham needles when they were shifted to the other needle (i.e., real needle to sham needle or vice versa). The feeling of skin penetrated was similar in Group A and Group B, which implied good concealment of using noninvasive sham needles. 

The study design limitation is the small sample size because no referred parameters are available from prior studies for healthy college students of Chinese medicine. If we use the VAS means and standard deviations as parameters for sample size calculation, in the significant level of *α* = 0.05 and power of 0.8 (*β* = 0.2), the required sample for each group is 61. Since healthy volunteers are difficult to recruit in clinical studies and the protocol compliance rate is 100% in this study, the data acquiring from this study provides valuable parameters for reference in future studies. Another limitation is that the study result is only appropriate to subjects with knowledge of Chinese medicine and acupuncture. The concept of deqi was not deconstructed into common life experience in this study; thus, it requires more profound understanding of acupuncture. The study participants were healthy college students; therefore, it cannot evaluate how deqi will affect the efficacy of acupuncture in real treatment. However, as deqi is considered an active factor for clinical efficacy, this study revealed that deqi is less likely to be evoked by noninvasive acupuncture, and tapping by sham needle on acupoints is felt even more painful than real acupuncture. These results will serve as valuable reference for future studies.

## Figures and Tables

**Table 1:** Baseline comparison of demographic data.

Comparison item	Group A (*n* = 30)	Group B (*n* = 30)	*P* value
Sex (%)			
Male	8 (26.7)	4 (13.3)	0.197*
Female	22 (73.3)	26 (86.7)	
Age			
Mean (SD)	23.13 (0.62)	22.93 (0.58)	0.201^#^

*Chi-square test, *χ* ^2^ = 1.667.

^#^Mann-Whitney test, *Z* = −1.277.

SD: standard deviation.

**Table 2:** VAS score in intervention Phase I and Phase II.

	Mean (SD)		*P* value
	Group A	Group B	
Between group comparison			
Phase I	1.43 (1.61)	2.33 (1.89)	0.053*
Phase II	2.60 (2.29)	1.99 (2.02)	0.282**
Within group comparison			
*P* value	0.017^#^	0.690^##^	

*Independent-samples *t*-test, *t* = 1.976. **Independent samples *t*-test, *t* = −1.087.

^#^Paired-samples *t*-test, *t* = −2.745. ^##^Paired-samples *t*-test, *t* = 0.580.

**Table 3:** ANOVA test for crossover effects.

Source of variance	*F*	*P* value
Treatment method	4.140	0.046
Treatment sequence	1.736	0.193
Subject	3.441	0.687

**Table 4:** Report of deqi feeling.

Perception of deqi	Group A (*n* = 30)	Group B (*n* = 30)	*P* value
Between-group comparison			
Intervention Phase I			
Yes	19 (63.3)	11 (36.7)	0.039*
No	11 (36.7)	19 (63.3)	
Intervention Phase II			
Yes	12 (40.0)	16 (53.3)	0.301**
No	18 (60.0)	14 (46.7)	
Within-group comparison			
*P* value	0.143^#^	0.332^#^	

*Chi-square test, *χ* ^2^ = 4.267, **Chi-square test, *χ* ^2^ = 1.071.

^#^McNemar test.

**Table 5:** Report of skin penetrated feeling.

Perception of skin penetrated	Group A (*n* = 30)	Group B (*n* = 30)	*P* value
Between-group comparison			
Intervention Phase I			
Yes	21 (70.0)	23 (76.7)	0.559*
No	9 (30.0)	7 (23.3)	
Intervention Phase II			
Yes	25 (83.3)	23 (76.7)	0.519**
No	5 (16.7)	7 (23.3)	
Within-group comparison			
*P* value	0.424^#^	1.000^#^	

*Chi-square test, *χ* ^2^ = 0.341, **Chi-square test, *χ* ^2^ = 0.417.

^#^McNemar test.
